# Glucagon-like Peptide-1 Receptor Agonist Treatment With Semaglutide in Type 1 Diabetes

**DOI:** 10.1210/jcemcr/luac017

**Published:** 2022-11-30

**Authors:** Lisa M Raven, Jerry R Greenfield, Christopher A Muir

**Affiliations:** Department of Diabetes and Endocrinology, St. Vincent's Hospital, Darlinghurst, NSW 2010, Australia; School of Clinical Medicine, St Vincent's Healthcare Clinical Campus, Faculty of Medicine and Health, UNSW Sydney, Kensington, NSW 2052, Australia; Clinical Diabetes, Appetite and Metabolism Laboratory, Garvan Institute of Medical Research, Darlinghurst, NSW 2010, Australia; Department of Diabetes and Endocrinology, St. Vincent's Hospital, Darlinghurst, NSW 2010, Australia; School of Clinical Medicine, St Vincent's Healthcare Clinical Campus, Faculty of Medicine and Health, UNSW Sydney, Kensington, NSW 2052, Australia; Clinical Diabetes, Appetite and Metabolism Laboratory, Garvan Institute of Medical Research, Darlinghurst, NSW 2010, Australia; Department of Diabetes and Endocrinology, St. Vincent's Hospital, Darlinghurst, NSW 2010, Australia; School of Clinical Medicine, St Vincent's Healthcare Clinical Campus, Faculty of Medicine and Health, UNSW Sydney, Kensington, NSW 2052, Australia

**Keywords:** type 1 diabetes, glucagon-like peptide-1 receptor agonist, GLP-1, semaglutide

## Abstract

The efficacy of glucagon-like peptide-1 receptor agonists in type 2 diabetes is well established, but their role in type 1 diabetes (T1DM) is less clear. A 36-year-old woman with a 27-year history of T1DM and undetectable c-peptide presented for review of weight management, with body mass index 29.3 kg/m^2^. A previous trial of dapagliflozin led to no improvement in weight or glycemic control. Semaglutide was introduced (0.25 mg weekly increased to 0.5 mg weekly) and was well tolerated. After 6 months, weight had decreased by 16 kg and insulin dose by 36%. Despite less insulin, hemoglobin A1c improved, with reduced glycemic variability and no increase in hypoglycemia. Semaglutide may exert significant metabolic benefits in patients with established T1DM, even where c-peptide is no longer detectable. This case supports the need for a dedicated trial examining potential benefits of semaglutide in T1DM.

The efficacy and safety of the glucagon-like peptide-1 (GLP-1) receptor agonist semaglutide in type 2 diabetes (T2DM) is well established. In patients with T2DM, semaglutide leads to weight loss, improvement in systolic blood pressure, and a reduction in composite cardiovascular outcomes [1]. In addition, higher doses of semaglutide are associated with weight loss in people with obesity without diabetes [2]. The role of semaglutide in patients with type 1 diabetes mellitus (T1DM) is less clear. The older GLP-1 receptor agonists, liraglutide, and short-acting exenatide, are associated with benefits in glycemic variability, insulin requirement and weight management in T1DM as add-on therapy to insulin [3, 4]. The GLP-1 receptor agonists act directly and indirectly on glucagon release and insulin resistance and have the potential to improve management in T1DM.

##  

### Case Presentation

A 36-year-old woman presented for review of T1DM. The patient described extreme difficulty in losing weight and persistent glycemic variability. She had been diagnosed 27 years earlier (aged 9 years). There were no known macrovascular complications; microvascular complications included stable, inactive proliferative diabetic retinopathy previously treated with bevacizumab and laser photocoagulation. Diabetes management included multiple daily injections of insulin, insulin glargine 300 units/mL 21 U daily and insulin lispro 16 U daily. Additional background history was significant for polycystic ovary syndrome (PCOS) treated with metformin 1000 mg twice daily and an oral contraceptive pill. Glucose monitoring was performed using the Freestyle Libre flash glucose monitoring system.

### Diagnostic Assessment

At the time of presentation, her weight was 78.8 kg and body mass index 29.3 kg/m^2^. Blood pressure was 137/86 mmHg. Hemoglobin A1c (HbA1c) was 7.2% (55 mmol/mol) with fasting c-peptide <0.05 nmol/L (<15.1 ng/dL; reference range [RR], 0.4-1.5 nmol/L). Urine albumin creatinine ratio was 3.3 mg/mmol (29.2 mg/g; RR, <3.5 mg/mmol). Creatinine was 70 µmol/L (0.79 mg/dL; RR, 45-90 µmol/L) with estimated glomerular filtration rate >90 mL/min/1.73 m^2^. Random total cholesterol was 4.8 mmol/L (185.6 mg/dL; RR, <5.6 mmol/L) with triglyceride 1.4 mmol/L (124 mg/dL; RR, <2.0 mmol/L).

### Treatment

With informed consent, there had been previous trial of a sodium glucose cotransporter-2 inhibitor, dapagliflozin 10 mg per oral daily. However, after 3 months, dapagliflozin had been ceased as there was minimal effect on glucose variability, no change in insulin doses, and no appreciable weight loss.

At the patient’s request, and with informed consent, treatment with semaglutide was initiated with a starting dose of 0.25 mg weekly subcutaneously. The dose was uptitrated to 0.5 mg weekly, which was associated with reduced appetite without significant nausea or gastrointestinal disturbance. Metformin 1000 mg twice daily was continued as management for PCOS. Retinal screening was performed before initiation of semaglutide to ensure retinopathy was inactive and stable compared with previous assessments.

### Outcome and Follow-up

After 6 months of treatment, the patient’s weight had decreased to 63 kg (−16 kg) and insulin doses were reduced by 43% (insulin glargine, −29%; insulin lispro, −63%). Systolic blood pressure reduced by 18 mmHg. Despite less insulin, HbA1c improved to 6.3% (−0.9%) with no increase in hypoglycemia. Changes in clinical and biochemical parameters before and after treatment with semaglutide are summarized in Table 1.

**Table 1.** Comparison of anthropomorphic and biochemical outcomes before and after 6 months of treatment with semaglutide 0.5 mg weekly

	Before semaglutide	After semaglutide	Net difference
Weight, kg	78.8	62.6	−16.2
Body mass index, kg/m^2^	29.3	23.3	−6.0
Systolic blood pressure, mmHg	137	119	−18
Diastolic blood pressure, mmHg	86	82	−4
Hemoglobin A1c, %	7.2	6.3	−0.9
Insulin dose, units	37	21	−16
Glargine (Toujeo)	21	15	−6
Lispro (Humalog)	16	6	−10

After 6 months of use, because of supply chain issues and inability to source semaglutide, her treatment was interrupted for 4 weeks, during which there was a deterioration in glycemia and increased glucose variability (Fig. 1).

**Figure 1.**
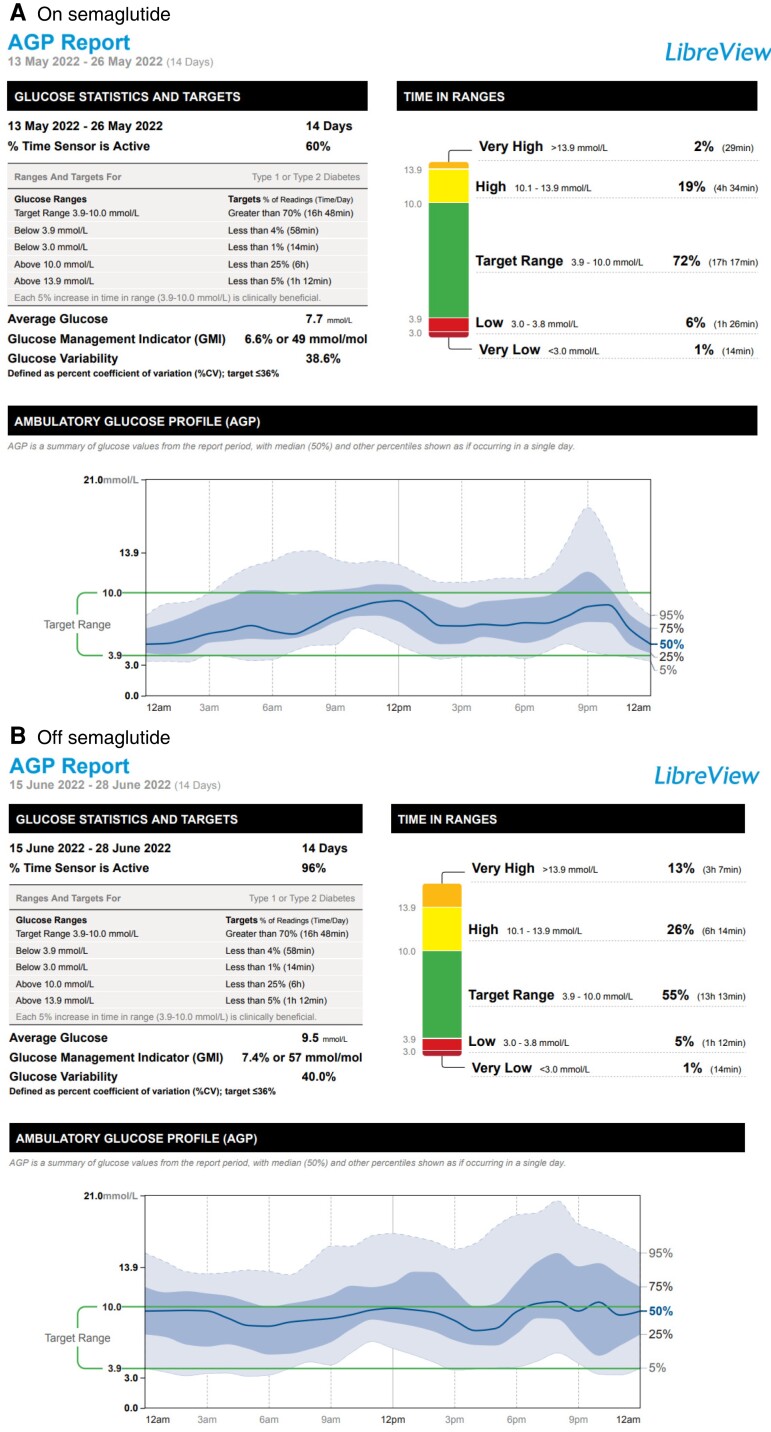
Glucose metrics on semaglutide with marked deterioration in glucose control back to pre-semaglutide baseline following withdrawal of semaglutide because of inability to source medications.

## Discussion

Insulin remains the mainstay of treatment for T1DM. However, adjunctive treatments such as the GLP-1 receptor agonists may provide additional therapeutic options through direct and indirect effects on glucagon release and insulin resistance.

T1DM is characterized by pancreatic α-cell dysfunction in addition to β-cell dysfunction [5]. Accordingly, there is impairment in glucagon secretion and release in addition to the better known defects in insulin secretion and release. Endogenous insulin suppresses glucagon secretion through its paracrine function. Exogenous insulin does not approach the paracrine levels of secretion from β cells and therefore is ineffective in suppressing glucagon secretion [5]. Hence, T1DM is characterized by postprandial hyperglucagonemia and hyperglycemia. GLP-1 receptor agonists suppress glucagon release, directly targeting hyperglucagonemia. Glucagon suppression may represent one mechanism underlying the therapeutic advantages observed with the addition of GLP-1 receptor agonists over insulin monotherapy.

Insulin resistance can also occur in T1DM by exogenous subcutaneous insulin injection bypassing first-pass hepatic insulin clearance, possibly by insulin receptor downregulation and/or promotion of abdominal adiposity through stimulation of lipogenesis [6]. In addition, there is IGF-1 deficiency, with secondary growth hormone hypersecretion, contributing to insulin resistance in T1DM [7]. GLP-1 receptor agonists indirectly improve insulin resistance by promoting weight loss. In the MAG1C trial, short-acting exenatide administered 3 times daily at mealtimes in people with T1DM was associated with a reduction in prandial insulin requirement and body weight, although there was no change in glucose levels or HbA1c [4].

There is conflicting evidence regarding residual pancreatic function on the effect of GLP-1 in T1DM. When ultrasensitive assays are used, most patients with more than 5 years of T1DM have detectable fasting c-peptide [8]. Furthermore, stimulated c-peptide levels after a mixed-meal tolerance test were detectable in more than 70% of this cohort [8]. A post hoc analysis of the exenatide trial in T1DM did not find any significant change when the cohort was stratified by fasting c-peptide levels above and below 30 pmol/L [4]. Conversely, in the largest study of liraglutide in T1DM, HbA1c improvement was limited to individuals with c-peptide ≥ 30 pmol/L in whom there was also a lower rate of symptomatic hypoglycemia and ketosis [3]. It is possible that the assays used in these studies and in our case do not detect microsecretion of insulin.

Patients with T1DM should be counselled about the potential adverse effects of GLP-1 receptor agonists. Gastrointestinal side effects of GLP-1 receptor agonists are well recognized, with dose escalation used to minimize the risk. Our patient tolerated semaglutide, in addition to metformin, without gastrointestinal side effects, which suggests that, as in patients with T2DM, patients with T1DM likely will experience a spectrum of gastrointestinal effects related to GLP-1 receptor agonists. Another consideration is the effect of GLP-1 receptor agonists, particularly semaglutide, on diabetic retinopathy. In T2DM, semaglutide 1.0 mg weekly was associated with deterioration in diabetic retinopathy, although this is deemed to be secondary to rapid glucose improvements rather than a direct effect of GLP-1 receptor agonists per se [1]. Because this issue remains an open area of investigation, retinal assessment should be undertaken before initiation of GLP-1 receptor agonists in patients with T1DM. GLP-1 receptor agonists, particularly shorter acting compounds, have powerful effects on gastric emptying and therefore may increase the risk of hypoglycemia in patients with T1DM. Hence, glucose levels and insulin requirements should be closely monitored following introduction of a GLP-1 receptor agonist because adjustments are likely to be required, particularly in prandial insulin doses, as in our patient (−63% in prandial and −29% in basal).

Our patient was overweight and had comorbid PCOS before initiation of semaglutide, a condition associated with increased body mass index and insulin resistance. GLP-1 receptor agonists, either as monotherapy or in combination with metformin, are associated with weight loss and improved metabolic markers in women with PCOS and overweight or obesity [9]. This is a potential additional benefit of GLP-1 receptor agonists in select patient groups and theoretically should not be restricted from use for treatment of PCOS or obesity based on a history of T1DM.

A higher dose of semaglutide, 2.4 mg weekly, has been associated with weight loss in people with obesity without diabetes [2]. Our patient used a reduced dose of 0.5 mg weekly, but still experienced substantial glycemic and metabolic benefits. In countries where 2.4 mg of semaglutide is not available (such as Australia), use of 1.0 mg has been frequently used off-label for obesity management. Because semaglutide has variable dosage options, it can be individualized to use the lowest effective dose.

Currently, there is only 1 report of semaglutide use in a patient with T1DM that demonstrated reduction in weight, insulin requirement, and HbA1c similar to reductions seen in our patient; that study did not report the c-peptide status of the patient [10]. Our case of semaglutide use in a patient with T1DM demonstrated metabolic benefits despite an undetectable fasting c-peptide at treatment initiation. Our data support the need for a dedicated trial examining potential benefits and risks of semaglutide in T1DM.

Author Contributions: L.R.: involved in manuscript writing and submission. C.M.: involved in the management of this patient and manuscript writing. J.G.: involved in manuscript writing. All authors reviewed and approved the final draft.

## Learning Points

- Insulin is the mainstay of treatment for type 1 diabetes; however, adjunctive therapies should be considered and further studied
- There may be metabolic benefits to the addition of glucagon-like peptide-1 receptor agonists in patients with type 1 diabetes
- The benefits of glucagon-like peptide-1 receptor agonists may not be limited to patients with detectable c-peptide levels
- The safety of glucose-like peptide 1 receptor agonists needs to be assessed further

## Data Availability

Data sharing is not applicable to this article as no datasets were generated or analyzed during the current study.

## Abbreviations

GLP-1: glucagon-like peptide-1
HbA1c: hemoglobin A1c
PCOS: polycystic ovary syndrome
T1DM: type 1 diabetes
T2DM: type 2 diabetes mellitus
