# Correction to “Glucagon-like Peptide-1 Receptor Agonist Treatment With Semaglutide in Type 1 Diabetes”

**DOI:** 10.1210/jcemcr/luac027

**Published:** 2022-12-28

In the above-named article by Raven LM, Greenfield JR, and Muir CA (*J Clin Endo Metab Case Reports*. 2023; 1(1): 10.1210/jcemcr/luac017), a production error occurred in the PDF version of Figure 1 regarding patient information.

The article has been corrected online.

The publisher regrets the error.

doi: 10.1210/jcemcr/luac017

