# Correction to “Glucagon-like Peptide-1 Receptor Agonist Treatment With Semaglutide in Type 1 Diabetes” *(J Clin Endo Metab Case Reports.* 2023; 1(1): 10.1210/jcemcr/luac017)

**DOI:** 10.1210/jcemcr/luad005

**Published:** 2023-01-24

A draft version of the correction notice for this article was mistakenly published. It has now been replaced with the final version. The Publisher apologizes for this error.

